# Policy analysis: Key milestones in MDR-TB management over the past decade in South Africa

**DOI:** 10.4102/jphia.v15i1.703

**Published:** 2024-12-17

**Authors:** Lee-Ann C. Davids, Talitha Crowley

**Affiliations:** 1 Office of AIDS and TB Research, South African Medical Research Council, Cape Town, South Africa; 2 School of Nursing, Faculty of Community and Health Sciences, University of the Western Cape, Cape Town, South Africa

**Keywords:** drug-resistant tuberculosis, management, milestones, multidrug-resistant tuberculosis, policy content analysis, policy review, rifampicin-resistant tuberculosis, South African National Department of Health, tuberculosis, World Health Organization

## Abstract

**Background:**

Significant strides have been made globally and in South Africa (SA) in the policy and biomedical management of multidrug-resistant tuberculosis (MDR-TB). However, MDR-TB remains a significant public health threat.

**Aim:**

This policy content analysis aims to explore the key milestones in MDR-TB management in SA and globally over the last decade, 2013–2023, to identify gaps and opportunities for improvement.

**Setting:**

This review focussed on global and South African national MDR-TB policies since SA is on all three World Health Organization (WHO) watch lists for TB, TB/HIV and MDR-TB, despite its significant contributions to policy.

**Method:**

A policy review and content analysis were conducted of all publicly available SA and WHO drug-resistant TB policies developed between 2013 and 2023.

**Results:**

Key changes identified were in the areas of new drug development and regimens, care delivery settings, task shifting and, terminology used in the field of drug-resistant TB. Changes in the biomedical sphere predominated in both SA and WHO policies.

**Conclusion:**

Important biomedical interventions have offered renewed hope for the SA MDR-TB programme. This review highlights that policy translation and implementation in non-biomedical interventions have been slow, sometimes lagging up to 10 years behind an intervention being recommended. This article recommends equal weight be placed on non-biomedical interventions and that these policy translations occur at a more rapid speed to positively impact the dire public health consequences of MDR-TB.

**Contribution:**

Insights offered through this policy review may contribute to policy development, translation and implementation towards improving MDR-TB outcomes in SA.

## Introduction

Tuberculosis (TB) is preventable and for the most part curable, however in 2023, TB very likely returned to its position as the leading cause of death globally from an infectious agent having been surpassed in the three years prior only by the novel SARS-CoV-2.^1^ Tuberculosis claimed an estimated 1.25 million lives in 2023 and affected millions more, with catastrophic impacts on families and communities^1^. In South Africa (SA), TB is the leading cause of death from an infectious agent, while multidrug-resistant TB (MDR-TB) remains a major public health threat with marked losses along each step of the care cascade, contributing to high morbidity and mortality.^2^

In 2021 the estimated TB incidence in SA was 513 per 100 000 population, approximately 300 000 TB cases and an estimated 57 317 persons succumbed to the disease^1^. Additionally, in the same year in SA there were an estimated 14 531 incident cases of MDR TB or rifampicin-resistant TB (RR-TB)^1^. The uncertainty interval for the number of reported MDR-TB and RR-TB cases for that period is also very wide.^1^ South Africa is among the 14 countries with the highest burden of MDR-TB and TB and HIV comorbidity.^1^ Treatment outcomes for patients with drug-resistant TB (DR-TB) in SA are generally poor. Approximately 60% of those on treatment are cured, while the average death rate is 17%, and the loss to follow-up (LTFU) rate stands at 17%.^3^

The second iteration of the South African TB recovery plan was released and implemented in 2023 to address these gaps in the care cascade.^3^ The goals of the SA TB recovery plan are aligned with those of the World Health Organization (WHO) End TB Strategy.^4^ South Africa has been a global pioneer in the field of RR-TB concerning diagnostics, novel treatments and policy.^5^ Yet, despite this, SA is on all three WHO global lists of high-burden countries for MDR/RR-TB, HIV-associated TB and TB.^1^

Mortality of persons with DR-TB in SA persists at an unacceptably high rate with recent data showing a 17% mortality rate among those initiating DR-TB treatment.^3^ This high mortality persists despite the implementation of numerous pharmacological and diagnostic interventions, disproportionately affecting the poor who often experience multiple afflictions. DR-TB mortality in SA is higher than in other African countries such as Nigeria, which has the highest TB burden on the continent and a mortality of 12%, 5 percentage points lower than SA.^6^ Despite the significant change in the availability of more effective and less toxic treatment regimens in SA accompanied by extensive global and local policy advancements to curb the burden of all forms of TB, mortality of MDR-TB remains high. Furthermore, disruptions in the health care system because of the coronavirus disease 2019 (COVID-19) pandemic may have caused nearly half a million excess deaths because of TB between 2020 and 2022 globally.^1^

The Global Project on Anti-TB Drug Resistance Surveillance has been in operation for the past 30 years (since 1994).^7^ Outbreaks involving strains resistant to two or more TB drugs were identified as early as the 1970s in New York.^8^ Locally, in SA, MDR-TB prevalence emerged in the 1980s with most of the resistance surveillance data being from the South African Medical Research Council.^9^ The TB and MDR-TB epidemics were fanned by HIV infection, especially in the era prior to the advent of widespread use and availability of anti-retroviral treatment.

The period between 2013 and 2023 was arguably the most significant in recent SA and global TB history. This period represents 10 years of significant policy change, conflicts and political changes locally in SA and internationally. The same decade also marked a dramatic evolution in the landscape of TB drug development, which had remained relatively stagnant for the four decades prior.^10^ During this period, bedaquiline, delamanid and pretomanid were approved. Additionally, the COVID-19 pandemic with its catastrophic sequelae on the TB programme and END TB targets occurred. The 2013–2023 decade ended with the roll-out of an all-oral four-drug MDR-TB regimen.

The introduction of bedaquiline and dolutegravir has simplified the management of those co-infected with HIV and TB. In fact, the approval of bedaquiline by the United States Food and Drug Association in 2012 can be considered a watershed moment in recent TB history as it was the first new anti-TB drug with a novel mechanism of action to be introduced in 40 years and it was approved without phase 3 trial data.^11^ Despite this key development, mortality and LTFU remain high at 17%. A high burden of DR-TB, significant DR-TB and HIV co-infection and substantial LTFU and mortality have been put forward as the main contributors to the DR-TB disease burden in SA.^12^

Multidrug-resistant tuberculosis remains a significant public health threat in SA despite advances in the biomedical sphere over the last 10 years, necessitating a review of policy measures to improve management, control and outcomes. Ismail et al.^13^ considered the gaps and opportunities of the current DR-TB epidemic in Africa and found that policy adoption and implementation of new diagnostic tools in Africa were lacking. This likely applies to the non-biomedical components of these policies too. Global MDR/RR-TB policies were significantly influenced by work carried out in SA;^5^ thus, a review of international policies must necessarily consider South African policies. There is a need to critically review and assess the key milestones and outcomes of MDR-TB policy interventions during this period to identify successes, shortcomings and areas for improvement. To this end, a policy content analysis of the South African National Department of Health and international WHO DR-TB policies over the last decade, 2013–2023 was conducted.

## Methods

### Design

**FIGURE 1:**
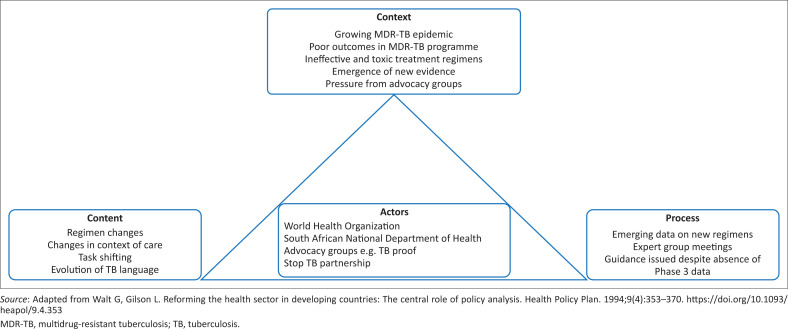
Policy triangle framework.

The health policy environment is a highly complex phenomenon which is influenced by a range of actors as well as local and global events.^14^ Policy analysis is two-pronged, comprising the analysis of the policy process and the analysis of the policy content.^14^ Walt and Gibson’s health policy analysis framework considers the complex interplay between policy content, the context, the actors and the policy process.^15^ This framework (see Figure 1) has been applied to the analysis of a broad range of issues, including tuberculosis.^15^ Applying this framework has been useful for this policy analysis as National TB Programmes (NTPs) are influenced by the context, processes and implementation strategies, actors and the content of the policies.

Although the broader aspects of policy analysis were considered, this review focussed on policy content analysis. Focussing on policy content analysis, rather than broader policy analysis, enabled a detailed examination of the language and frameworks within MDR-TB policies. This targeted approach revealed the themes and priorities that shape policy implementation, essential for addressing the unique challenges of managing MDR-TB in SA.

**FIGURE 2:**
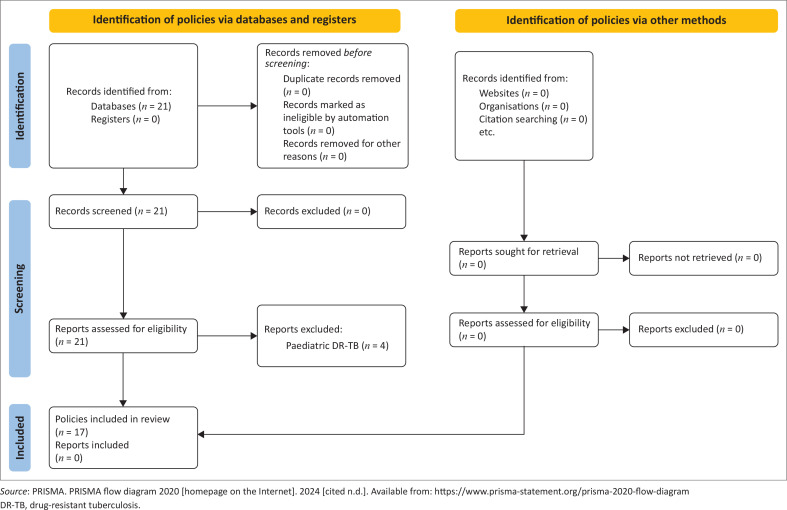
PRISMA flow diagram of record indentification and selection process.

Collins provides a framework that allows for the comprehensive analysis of policy content.^16^ This framework is composed of the following eight steps: namely ‘(1) define the context; (2) state the problem; (3) search for evidence; (4) consider different policy options; (5) project the outcomes; (6) apply evaluative criteria; (7) weigh the outcomes and (8) make the decision (p. 194)’.^16^ The framework is particularly useful for this study’s research question as it offers guidance on how to ‘… analyse policy and link it to health outcomes (p. 192)’.^16^ In the ‘Introduction’ the article provides the context and states the problem. The ‘Methods’ section outlines the data sources and search strategy, as well as the data extraction and analysis, demonstrating how the search for evidence was conducted and how various policy options were evaluated through a review of SA and WHO policies, and relevant advocacy partner policies. The ‘Results’ section, which includes Figure 2^17^ and Table 1, presents the outcomes and illustrates the evaluative criteria that were applied. In the ‘Discussion’ and ‘Conclusion’, the outcomes are assessed, and recommendations are provided, respectively.

**TABLE 1:** List of policies reviewed.

Year of policy	Policy name
2013	World Health Organization: The use of bedaquiline in the treatment of multidrug-resistant TB – Interim policy guidance
	South African National Department of Health: Management of drug-resistant TB – Policy guidance
2014	World Health Organization: Use of delamanid in the treatment of multidrug-resistant TB – Interim policy guidance
	World Health Organization: Companion handbook to the World Health Organization guidelines for the programmatic management of drug-resistant TB
	South African National Department of Health: National TB management guidelines
2015	South African National Department of Health: Introduction of new drugs and drug regimens for the management of drug-resistant TB in SA – Policy framework
2016	World Health Organization: Treatment guidelines for drug-resistant TB – 2016 multidrug-resistant TB
	World Health Organization: Fact sheet on the shorter multidrug-resistant TB regimen
2018	World Health Organization: Rapid communication; Key changes to the treatment of multidrug – and rifampicin-resistant tuberculosis
	World Health Organization: Position statement on the use of delamanid for multidrug-resistant TB
	South African National Department of Health: Interim clinical guideline for the implementation of injectable-free regimens for rifampicin-resistant TB in adults, adolescents and children
2019	World Health Organization: Consolidated guidelines on drug-resistant TB treatment
	South African National Department of Health: A policy framework on decentralised and deinstitutionalised management for South Africa
2020	World Health Organization: Consolidated guidelines on TB; Module 4: Treatment of drug-resistant TB
2022	World Health Organization: Rapid communication: Key changes to the treatment of drug-resistant TB
	World Health Organization: Consolidated guidelines on TB; Module 4: Treatment of drug-resistant TB – 2022 update
2023	South African National Department of Health: Clinical management of rifampicin-resistant TB– Updated clinical reference guide

SA, South Africa; TB, tuberculosis.

### Data source and search strategy

A policy review and content analysis of all publicly available South African and WHO DR-TB policies were conducted. To provide a comprehensive review, all WHO and South African DR-TB policies between 2013 and 2023 were considered as this was the period of the most drastic changes in the DR-TB programme internationally and locally and marked a dramatic evolution in the landscape of TB drug development, which had remained relatively stagnant for the four decades prior.^10^ Online searches were performed on Google, the WHO official website, the SA National Department of Health (NDOH) website, Knowledge Hub (SA professional development platform) and important advocacy group websites such as the Stop TB Partnership.

The search was conducted between November 2023 and January 2024. This was to ensure that the data were comprehensive and current for the period under study. The search terms ‘multidrug-resistant’, ‘rifampicin-resistant’, ‘Tuberculosis’ and ‘South Africa’ were used and the applicable years. Policies were included if they were: (1) written in the English language, (2) included content related to MDR-TB programmes and management, (3) were published between 2013 and 2023, (4) contained significant changes related to the DR-TB programme and (5) were either WHO or SA NDOH policies. Documents and policies that only included paediatric management were excluded because the focus of this policy analysis was on adults. The document identification and selection processes are indicated in Figure 2. Once the search was completed, a list of policy documents was collated and placed chronologically (Table 1). In total, 11 WHO and six SA NDOH policy documents were reviewed. All the documents were publicly available and easily accessible.

### Data extraction and analysis

The policies were read and re-read several times to achieve full comprehension and using conventional qualitative content analysis, a matrix (Appendix 1) was developed as key themes emerged.^18^ In this method, codes are derived as they emerge from the data.^18^ This process was repeated until saturation was achieved. The authors discussed the codes on several occasions to determine the relevance to the research aim and to reach a consensus. The authors critically analysed the evolution of these policies, tracking key changes at various junctures, and examined how the approval of new pharmacological agents influenced policy changes over time. Key changes occurred in the areas of biomedical interventions, including new diagnostics and drug regimens, as well as in the settings of care delivery and task shifting. Issues pertaining to patient-centred care and non-pharmacological support featured less prominently.

The final review of the documents and completion of the matrix occurred concurrently. For all the policies included in the matrix, data were organised into the following column headings:

- Year of document (when the policy was published)
- Type of document and actors – SA NDOH or WHO (policy actors)
- Context (Emerging evidence or other significant events that influenced the policy change)
- Context of care (centralised vs. decentralised care recommended)
- Care provider (Who the primary health care provider is)
- Regimen (recommended regimen or changes)
- Language used (punitive vs. patient-centred in keeping with the Stop TB partnership guidelines on TB terminology).^19,20^

Once the matrix had been completed, the authors synthesised the data into a narrative.

### Ethical considerations

No ethical approval is required for this review, as it relies on publicly available, published data and does not involve human participants.

## Results

In total, 21 policies were identified and 17 DR-TB policies which feature key milestones over the last decade were included (Figure 2). Of these, 11 were WHO documents and six were SA NDOH documents (Table 1). In addition, the 2024 Global TB Report was reviewed as well as the two Stop TB Partnership documents on TB terminology.^1,19,20^ The documents encompassed a broad spectrum, ranging from fact sheets to rapid communications and guideline updates. The authors are of the opinion that this provided sufficient data for a comprehensive analysis.

Milestones and key MDR-TB management focus areas:

- Introduction of novel drugs and shorter regimens leading to the bedaquiline/pretomanid/linezolid+/-moxifloxacin regimen
- Changes in the context of care – centralised vs. decentralised
- Task shifting from medical doctor to trained clinician for example nurse or clinical associates
- Transitioning towards patient-centred language use.

For simplicity and clarity, the guidelines are presented in Table 1 in chronological order from 2013 to 2023 for both the SA NDOH and WHO. New drug development and the introduction of shorter regimens have been the dominant changes over the last decade in the field of MDR-TB and as such, this write-up was skewed towards these developments.

### Area 1: Introduction of novel drugs and shorter regimens leading to bedaquiline/pretomanid/linezolid+/-moxifloxacin

The 2013 SA NDOH guidelines recommended a standardised approach to the management of persons with MDR-TB consisting of an intensive phase which included an injectable for at least 6 months and a continuation phase of 18 or less months.^21^ The drugs used during the intensive phase included the injectable kanamycin or amikacin, moxifloxacin, ethionamide and terizidone. During the continuation phase moxifloxacin, ethionamide, terizidone or cycloserine and pyrazinamide were used. The aminoglycoside group of drugs formed the backbone of therapy. This was in line with WHO guidance.^22^

The 2013 SA NDOH guidelines on the management of DR-TB did not make mention at all of bedaquiline. Yet, in 2013, the WHO released an interim policy guideline on the use of bedaquiline in the treatment of MDR-TB.^22^ This was after a meeting with an international expert group which was held on 29 January 2013–30 January 2013 at the WHO in Geneva.^22^ This meeting was convened following the approval of bedaquiline as member states were seeking guidance. Although this was based on Phase IIb trial data only, the WHO asserted that interim guidance was necessary for the responsible use of bedaquiline to avoid resistance.^22^

When bedaquiline received accelerated approval from the United States Food and Drug Association in 2012, it was the first new anti-TB drug in 40 years to be approved with a novel mechanism of action; the approval of rifampicin was in 1974.^11^ This was a watershed moment in recent TB history. It signalled the end of the dearth of new TB drug development and heralded an era of rapid development of new TB drugs and in particular, drugs effective against DR-TB strains. In December 2012, the SA Medicines Control Council approved a bedaquiline clinical access programme (BCAP) for SA.^23^ Experience gained through this clinical access programme informed the SA NDOH 2015 guideline on the use of bedaquiline in the NTP.^24^ There was thus a 2-year lag between the time the WHO released guidance and SA released its own. South Africa’s approach could thus be described as astute and cautious, and consideration must be given to whether this delay may have influenced the outcomes of the DR-TB programme in SA.

In April 2014, the WHO again convened an expert group meeting to discuss the use of delamanid.^25^ Delamanid, another new compound, had been granted authorisation conditionally to be marketed by the European Medicines Agency Committee for Medicinal Products for Human Use.^25^ Subsequent to these developments, the WHO released interim policy guidelines for the conditional use of delamanid for DR-TB.^25^ As for bedaquiline, this decision was taken in the absence of phase III trial data.^25^ Delamanid was to be used with a WHO approved regimen.

In 2014, WHO guidelines had already made provision for shorter regimens ranging from 9 to 12 months but cautioned that evidence for these shorter regimens was limited and only one study, namely the Bangladesh study, had been published and peer-reviewed.^25^ The recommended total treatment duration, however, was still 20 months. The WHO 2014 guideline^26^ incorporated key changes in drug development and considered policy changes with regard to bedaquiline and delamanid. Though this policy can be viewed as a landmark development as it considered significant developments in diagnostics and new drugs, it acknowledged that diagnostics and new drugs alone fall short of addressing the complexities of MDR-TB management.^26^ The 2014 SA NDOH policy also recommended 18–24 months of treatment but it did not elaborate on which specific drugs should be given.^27^

In 2015, the SA NDOH recommended that eligible patients should have bedaquiline added to an optimised background regimen and that delamanid will be introduced in 2015 through a phased approach.^24^ The price of delamanid was however prohibitive. On World TB Day in 2017, a delamanid clinical access programme was launched which was anticipated to benefit at least 400 patients with MDR-TB in SA.^28^ At this time, DR-TB treatment success was below 50%.^24^ Injectables were also still part of the standard treatment regimen which was to be 18–24 months post culture conversion.^28^ In 2016, two new drugs, linezolid and clofazimine were recommended as part of the core second-line therapies.^29^ In this same year, the WHO introduced a 9–12 month standardised shorter MDR-TB regimen; this regimen, however, still included an injectable during the intensive phase of treatment.^30^

From 2016 to date (2024), the treatment landscape developed dramatically with the addition of new and repurposed TB chemotherapeutics to the treatment arsenal. These include clofazimine, pretomanid, linezolid, protionamide and para-aminosalicylate sodium. Policy changes also occurred more rapidly to keep abreast with the fast emergence of new evidence.^31^ Increasingly pressure was mounting from the medical community and advocacy groups for injectables to be removed from the MDR-TB treatment regimen, for the use of newer and more effective drugs to be included in the treatment regimen and for treatment duration to be shortened.

In 2018, the SA NDOH slowly started phasing in injection-free regimens as part of the short course regimens for uncomplicated MDR-TB and all provinces were informed of a plan to expand the use of bedaquiline.^32^ Between 2018 and 2020, both the WHO and SA NDOH introduced various iterations of short-course regimens and long-course regimens with and without injectables including new and repurposed drugs as they were approved by the various regulatory authorities and as trial results were released. In 2020, the WHO Consolidated guidelines on TB^33^ recommended an all-oral regimen of 6 to 9 months.

The most dramatic change in the pharmaceutical treatment regimen of MDR treatment was implemented in 2022. Data from the ZeNix, Next and TB Practecal trials became available and informed the introduction of the all-oral 6-month bedaquiline, pretomanid, linezolid and moxifloxacin (BPaLM) regimen.^34^ This change was released via rapid communication from the WHO^34^ and was influenced by fears that the COVID-19 pandemic could result in increased TB incidence. To incorporate this change and to facilitate policy transfer at a country level, the WHO released updated consolidated guidelines which made provision for the 6-month BPaLM regimen for MDR and Pre-XDR-TB but the moxifloxacin should be dropped if fluoroquinolone resistance is detected.^35^ South Africa made a significant contribution to these guidelines with their programmatic and trial data.^35^ In 2023, SA followed suit and released guidelines for the implementation of the all-oral 6-month BPaL/L regimen for MDR-TB.^36^

### Area 2: Changes in the context of care – Centralised vs. decentralised

In 2013, the WHO released an interim policy on the use of bedaquiline but no mention was made about where care should be delivered.^22^ This is interesting given the significant cardiovascular changes associated with bedaquiline or it may be that the WHO was delegating the responsibility of deciding on where care should be delivered to individual countries. In 2013, the SA NDOH recommended that each province have a centralised DR-TB unit. This unit would be referred to as a ‘Provincial Centre of Excellence’ and could be a stand-alone hospital or a dedicated DR-TB ward in a general, specialised or TB hospital.^21^ This hierarchical arrangement was a centralised unit, decentralised unit, satellite MDR-TB unit, mobile and primary health care teams and community support workers. Treatment was to be initiated in the hospital only; however, the policy cautioned that treatment initiation should not be delayed because of patient refusal to be admitted or bed unavailability.^21^ Patients were to be hospitalised for up to 8 weeks or until they had two smear-negative sputum microscopy results. The centralised unit also played an oversight role and had to monitor patients for 2 years after they had completed treatment. Though the care was centralised, decentralisation was offered as a solution for patients who refused hospital admission or requested premature discharge while still infectious.

The same applies to the 2014 WHO interim policy on the use of delamanid which did not specify where care should be delivered.^25^ The WHO companion handbook of the same year however recommended ambulatory models of care rather than hospital-based care but cautioned that there was low-quality evidence to support this conditional recommendation.^26^ Furthermore, the WHO^21^ advised that no single model of care would be able to address the needs of all patients and that a combination of models would likely be required. In 2014, the South African National Tuberculosis Management guidelines^27^ recommended that all persons with MDR-TB be hospitalised until they were proven non-infectious. This recommendation was so harsh that hospital admission could be enforced with a court order.^27^ Post-discharge ongoing treatment could be provided at primary health care facilities. Additional safety monitoring is required for patients on bedaquiline and therefore the 2015 SA NDOH guidelines recommended either in-patient or out-patient management of patients on bedaquiline depending on the local capacity for implementation and monitoring.^24^

The WHO 2016 DR-TB updates,^29^ 2018 rapid communication on key changes to the MDR-TB guidelines^31^ and the 2019 consolidated guidelines^37^ all recommended ambulatory or decentralised care. It was only in 2019 that the SA NDOH released a framework for the decentralisation and deinstitutionalisation of MDR-TB care.^38^ However, in the years prior, the number of decentralised sites had been on the increase and the period of stay in the centralised sites had slowly been reduced. This policy provided for out-patient initiation and further management of persons with MDR-TB provided they were in a ‘… fair and general condition…’ (p. 11).^38^ Each sub-district was to have at least one MDR-TB treatment initiation site. This policy made provision for the delivery of care through various modalities depending on the patient’s needs – including in-patient or out-patient delivery methods. Importantly, where previously admission was dictated by the patient’s microbiological status, it was now determined by the patient’s condition. This marked a significant shift in patient care and points increasingly to a patient-centred approach to care delivery. This policy decision was inspired by the increase in treatment success, other benefits observed for persons on short and long-course regimens and the infeasibility of hospital admission.^38^ All subsequent WHO and SA NDOH policies to date recommended a decentralised approach.

### Area 3: Task shifting from medical doctor to trained clinician for example nurse or clinical associates

As early as 2013, the SA NDOH recognised that the significant burden of MDR-TB could be addressed by Nurse-Initiated Management of MDR-TB treatment (NIMDR). At this time, treatment initiation was the sole responsibility of medical practitioners (doctors). The NIMDR argument was further strengthened by the success of the Nurse Initiated Management of Anti-retroviral Treatment (NIMART) programme globally.^26^ Nurses largely had a coordination and support function in the management of persons with MDR-TB.

The WHO 2014 DR-TB Companion Handbook^26^ recommended an experienced MDR-TB physician perform the initial assessment though it acknowledged the importance of nurses in the multi-disciplinary team. The 2016 WHO update of the same guideline made no reference to who should initiate treatment.^29^ The 2019 WHO Consolidated MDR-TB guidelines^37^ did not specifically mention who should initiate treatment but reviewed data that proved decentralised care could be provided by non-specialised doctors, nurses and others which differs from the 2014 WHO guidelines that recommended the initial assessment be carried out by an experienced MDR-TB physician.

There was a significant shift in SA policy in 2019^38^ which saw nurses and clinical associates playing a more central role in patient management. This policy made provision for the establishment of one treatment initiation site per sub-district which could be managed by a clinical nurse practitioner, a medical officer or a clinical associate. The inclusion and more central involvement of nurses and clinical associates was considered essential to the success of decentralisation. The shift in policy is noteworthy as it sees nurses moving from a support role to an overall leadership role. Medical doctors were still responsible for initiating treatment both at the centralised and decentralised units, but a large portion of the ongoing management and care was to be delegated to nurses.

Nurse-Initiated Management of MDR-TB treatment was incorporated into South African policy in 2023.^36^ Thus, a full decade had passed since the recommendation that this initiative could assist in addressing the significant burden of MDR-TB. The policy provided that trained nurses should continue initiating treatment of persons with uncomplicated MDR-TB. It further highlighted that data showed comparable outcomes between patients who were initiated on treatment by nurses versus those who were initiated by doctors.^36^ There is an ongoing cluster randomised, non-inferiority trial comparing nurse-led treatment of RR-TB with physician-led treatment management.^39^ This trial is being conducted in rural settings in the Eastern Cape and KwaZulu Natal provinces and findings from this trial will be critical, especially in underserved/understaffed areas which are generally nurse-run.

### Area 4: Move towards patient-centred language use

The terminology used in the field of TB had for decades been punitive and implied blame. It is well documented how detrimental the use of such language has been to patients and indeed the TB programme.^35^ The language we use shapes our paradigm and care for persons with TB. For example, the 2013 SA DR-TB guidelines refer to DR-TB as a ‘man-made problem largely due to human error’ (p. 4).^21^ Subsequent policies have made a similar assertion. The words ‘TB treatment defaulter’ and ‘TB suspect’ formed part of the everyday TB language among health care workers.^33^ The stigmatising impact of such harmful TB terminology was raised at a meeting convened by the International Union Against Tuberculosis and Lung Disease (The Union) and Médecins Sans Frontières in Paris and prompted Zachariah et al.^40^ to write to the STOP TB Partnership to take a leading role towards changing the language used in the field of TB. In response to this appeal, the STOP TB Partnership developed a ‘living document’ in 2015 named Every Word Counts which was aimed at developing more patient-centred language to lessen the harmful impact of current TB terminology.^19^ The authors emphasised that this document is likely to evolve as language evolves. Notably, the term *compliance* was changed to *adherence, default* was changed to *lost to follow-up* and the *person with TB symptoms* was preferred to *TB suspect.*^19^ It is worth noting that in 2014, prior to the release of this guideline, the WHO had already begun emphasising the need for change to more empowering patient-centred language.^26^ Though they suggested using words like *LTFU* as opposed to *default*, it was not applied uniformly throughout the guideline.^26^ This may be because the 2014 WHO guidelines were published prior to the release of the Every Word Counts document in 2014.

The second edition of this document published in 2022 was named Words Matter and included wider collaboration with various sectors including persons with lived experiences.^20^ The authors emphasised that language shapes our worldview and influences whether someone who likely has TB will engage with the health care service. Updated terminology was more patient-centred and gender sensitive. It also made a distinction between which words should not be used and which words should be used with caution. It included a section on TB classification as new knowledge about the continuum of TB infection became available.

## Discussion

Over the past decade, key milestones and focus areas in MDR-TB management have included the introduction of novel pharmacological agents to shorten treatment duration and reduce side effects, the decentralisation of care, task shifting and the use of patient-centred language.

The introduction of bedaquiline was a watershed moment in DR-TB history even though its use was approached with many caveats. The expert group of the WHO acknowledged that it was novel for the WHO to develop guidelines for new drugs based on Phase IIb trial data only.^22^ This decision, however, was necessitated by the very low cure rate, high LTFU and high mortality among patients with MDR-TB.^22^ Treatment was long, arduous, toxic and expensive, which necessitated a different approach. South Africa’s approach to the introduction of novel agents and regimens was, however, conservative, which may have hampered the progress of the MDR-TB programme.

**FIGURE 3:**
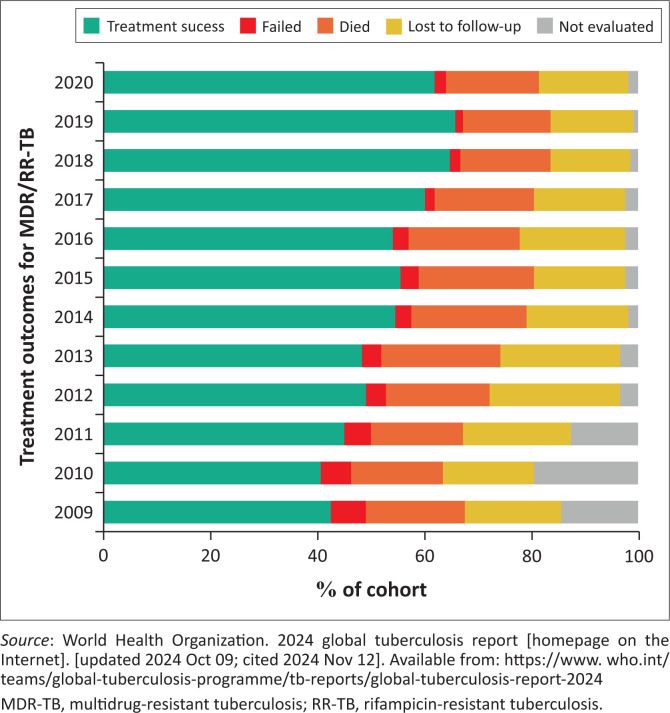
Multidrug-resistant tuberculosis outcomes for South Africa since 2009.

The current DR-TB regimen being recommended by the WHO globally and locally was informed by research and programmatic data from SA.^23^ It is estimated that only around 60% of persons on MDR-TB treatment in SA are cured, and this is despite the dramatic evolution of the DR-TB diagnostic and therapeutic landscape both locally and globally in recent history.^1^ This is particularly concerning given that in 2014, treatment success rates were reported to be below 50%, and the National Strategic Plan at that time aimed for a treatment success rate of 60%.^41^ In other words, SA has only just surpassed the goal for treatment success that was established a decade ago despite dramatic biomedical advances in the DR-TB programme. There is thus a significant opportunity for improving outcomes of DR-TB as evidenced by Figure 3^1^. From this figure, it appears the decline in mortality and loss to follow up has been modest. Understanding why the very same innovations that influenced WHO policies have not improved treatment outcomes in South Africa is an important question to address. This policy content analysis provides an evidence base for this inquiry.

All current WHO and SA NDOH policies recommend a decentralised model of care for persons with MDR-TB. In 2014,^21^ the WHO already acknowledged the key role that ambulatory models of care could play but it was only in 2019 that the SA NDOH released a framework for the decentralisation of MDR-TB care.^38^ This represented a significant leap from SA’s 2014 guidelines^27^ where hospitalisation could be enforced with a court order. It does, however, represent a significant delay in the implementation of best practices. Nurse-Initiated Management of MDR-TB treatment was offered as a potential solution to the burden of MDR-TB in 2013^21^ and incorporated into policy in 2023^36^ representing a 10-year period from the time a potential solution was identified to when it was incorporated into policy. Learning from the NIMART programme, it is reasonable to conclude that significant strides in the SA MDR-TB programme could have been made had this intervention been introduced earlier.

From this review of the policies and guidelines over 10 years, since 2013, a patient-centred approach has been an aspiration of both the WHO and SA NDOH. The WHO 2015 End TB Strategy lauded patient-centred care as the first pillar towards achieving the 2035 End TB targets.^4^ From the analysis, language has a direct bearing on how we see and care for persons with TB. The Stop TB Partnership guidance^19,20^ has made significant strides in helping the TB world use language that will encourage patients to engage with the health care system which may lead to improved outcomes.

Providing patient-centred care was a noticeable gap in this review. Myburgh et al.^42^ write that despite the WHO’s acknowledgement of the importance of patient-centred care, attempts to control TB have largely discounted the experiences of those affected by the disease. The authors highlight the complexities involved in integrating patient-centred care into TB programmes and conducted a scoping review to assess how these approaches have been incorporated.^42^ Their findings indicate that only a limited number of interventions have been implemented.^42^ To this end, much more research and importantly, implementation is required. In this policy content analysis, patient-centredness was an aspiration; however, no clear guidance on implementation was provided, nor was there any evidence that it was actually achieved.

## Conclusion

The roll-out of the all-oral MDR-TB regimen, BPaLM, along with new diagnostics for early TB detection and advanced digital adherence technologies, offers renewed hope for enhancing MDR-TB programmes and improving patient outcomes. However, biomedical interventions alone are unlikely to enhance patient-centred care and achieve the desired results. This policy content analysis provides an evidence base for incorporating a biopsychosocial approach into TB programmes while leveraging existing biomedical interventions to improve outcomes. Furthermore, the analysis highlights significant delays, often lasting up to a decade, between recognising the importance of an intervention and its implementation. These missed or delayed opportunities are detrimental to TB programmes and need to be addressed.

## Appendix 1

**TABLE 1-A1:** Data analysis matrix.

Year	Document	Context	Context of care	Care provider	Regimen	Language
2013	SA NDOH. Management of DR-TB. Policy guidance. Updated January 2013	No guidance yet on the use of Bdq	Largely centralised but decentralisation offered as solution	Doctor-initiated	Aminoglycoside-containing. 18–24 months. Standardised regimen only in persons not previously exposed to second-line TB drugs	Punitive
2013 June	WHO Interim guidance on use of Bdq following the expert group (EG) meeting	Bdq approved with only Phase IIb trial data	Not mentioned	Doctor-initiated	Bdq + WHO recommended regimen under certain conditions only	Patient-centred
2014	WHO – The use of Dlm in treatment of MDR-TB – Interim policy guidance	Conditional approval granted by expert group. No phase III data available	Not mentioned	Not mentioned	Dlm + WHO approved regimen	Punitive
2014	Companion Handbook to WHO guidelines for programmatic management of DR-TB	Rapid emergence of new evidence pertaining to drug development incorporated in this guideline	Ambulatory rather than principally hospitalisation	Doctor-initiated	Provision made for 9–12-month short course regimen (Scr). 5 – Group system + injectable. Group 5 – which included Bdq and Dlm not recommended for routine use	Patient-centred
2014	SA NDOH. National TB management guidelines	New rapid diagnostic tests introduced but culture still used as confirmation	Centralised. Discharge once non-infectious	Doctor-initiated	18–24 months but does not elaborate on which regimen or drugs to use	Punitive
2015	SA NDOH. Introduction of new drugs	Info for how National TB Programmes will introduce Bdq based on experience gathered through Bedaquiline Clinical Access Programme (BCAP). Treatment success below 50%	Patients on Bdq to be managed as in-patient or out-patient depending on local guidelines and capacity	Doctor-initiated	18–24 months post culture conversion. First time patients receive standard regimen with injectable	Patient-centred
2016	WHO-Treatment guidelines for DR-TB – 2016 Update	Replaces 2011 recommendations. Medicine reclassification. Rapid DNA-based test such as Genotype recommended as initial test	Ambulatory preferred to hospitalisation	Doctor-initiated	18–24 months including injectable. Scr in select cases (including injectable). Clofazimine (cfz) and Linezolid (Lzd) recommended as core second-line medicines	Patient-centred
2016	WHO Fact Sheet on the shorter MDR-TB regimen	Based on WHO updated 2016 guidelines	Not mentioned	Not mentioned	4–6 months km, mfx, pto, cfz, z, Hd inh and e; 5 months mfx, cfz, z, e. Failing regimen, drug intolerance, return after interruption > 2 months, emergence of any exclusion criterion → individualised (‘conventional’) MDR/RR-TB regimen	Patient-centred
January 2018	WHO Position Statement on Dlm	Phase III trial data on Dlm + Optimised Background Regimen (OBR) available. Expedited review done	Not mentioned	Not mentioned	As for 2016 with conditional addition of Dlm. Dlm can only be added to longer regimens, not to Scr	Not mentioned
August 2018	WHO Rapid communication: key changes to MDR and RR-TB guidelines	New evidence assessment of relative contribution of individual medicines	Ambulatory	Doctor-initiated	All newly diagnosed start at 9–12 months. Injectables no longer recommended	Patient-centred
2018	SA NDOH. Interim clinical guidance for the implementation of injectable-free regimen for RR-TB for adults and children	Injection-free regimens being phased in. ‘Bdq expansion plan’ circulated to all provinces	Not mentioned	Clinician-initiated	Scr of 9–11 months: Lzd, Bdq, lfx, cfz, Hd inh, z, e. For uncomplicated MDR or RR-TB and uncomplicated Extra pulmonary TB. Long course regimen (Lcr) of 18–20 months: Lzd, Bdq, lfx, cfz, trd	Patient-centred
2019	WHO Consolidated guidelines on DR-TB treatment	Incorporates various treatment guidelines between 2011 and 2018	Decentralised	Clinician-imitated	Scr 9–12 months for previously untreated. All-oral regimens recommended. Longer regimens – start with all 3 group A agents and at least one Group B	Patient-centred
2019	SA NDOH. Policy framework on decentralised and deinstitutionalised management of MDR-TB	The need for and benefits of decentralisation and deinstitutionalisation	Decentralised. All RR/MDR-TB patients in fair to good general condition may be started on ambulatory treatment regardless of their bacteriological status	Site may be led by Medical Officer (MO), clinical associate or NIMDR-trained clinical nurse practitioner	N/A	Language is punitive but approach to where care is delivered is said to be patient centred
2020	WHO. Consolidated guidelines on TB. Module 4-DR-TB treatment	Evidence-based info to inform all-oral regimens especially BPaL for MDR/RR-TB and persons with FLQ resistance	Decentralised model of care recommended over centralised	Not mentioned	Scr: 4–6 lfx/mfx, cfz, z, e, Hd inh, eto and 5 months lfx/mfx, cfz, z, e. Scr now recommended as a standard package	Patient-centred
May 2022	WHO. Rapid communication: Key changes to the treatment of DR-TB	Impact of the COVID-19 pandemic, fears that TB incidence could increase in 2022 and 2023 globally. Availability of data from TB Practecal, ZeNix and NeXT trials	Not specified but presumably decentralised	Not mentioned	6/12 BPaLM if no fluoroquinolone resistance may be used programmatically	Patient-centred
2022	WHO Consolidated TB guidelines	In response to requests from Member States, to facilitate policy transfer at the country level. 1st iteration was in 2019. Incorporates 2022 rapid communication. Supersedes 2020 guidelines. SA programmatic and trial data provided a significant contribution	Not specified but presumably decentralised	Not mentioned	6/12 BPaLM for MDR/RR-TB. Drop mfx if resistant to flq. For all over 14 years. Lzd dose 600mg. If not eligible for 6-month regimen, use 9-month regimen	Patient-centred
2023	SA NDOH. Clinical Management of Rifampicin-Resistant Tuberculosis	Aligns V2.0 of TB recovery plan with National Strategic Plan (NSP) for 2023–2028 which aims to address gaps in treatment cascade following the COVID pandemic. Based on the most recent WHO recommendations. Supports implementation of BPaLM	Decentralised	Nurse Initiated management of MDR-TB (NIMDR)	6-month BPaL/L	Patient-centred

Notes: 2012, December: Bedaquiline (Bdq) receives accelerated approval from the United States Food and Drug Association (FDA) for MDR-TB together with an approved background MDR-TB regimen 2013, January 29 – 30: International Expert Group (EG) Meeting in Geneva, Switzerland. South Africa representative formed part of the EG. 2014, April: Expert group meeting held 15 – 16 April 2014. South Africa representative formed part of the meeting delegation. The recommendation was that delamanid (dlm) (100 mg twice daily for 6 months) may be added to a WHO-recommended regimen in MDR-TB adult patients under specific conditions. 2014: Dlm, a nitro-imidazole, another new compound, has been granted a conditional marketing authorisation by the European Medicines Agency (EMA) Committee for Medicinal Products for Human Use (CHMP) on 28 April 2014.

Bdq, bedaquiline; Dlm, delamanid; BCAP, Bedaquiline Clinical Access Programme; DCAP, Delamanid Clinical Access Programme; Lzd, linezolid; km, kanamycin; mfx, moxifloxacin; Pto, prothionamide; cfz, clofazimine; z, pyrazinamide; Hd inh, High-dose-Isoniazid; e, ethambutol; BPaLM, bedaquiline/pretomanid/linezolid/moxifloxacin; BPaL/L, bedaquiline/pretomanid/linezolid/levofloxacin; SA, South Africa; MDR-TB, multidrug-resistant tuberculosis; NDOH, National Department of Health; FLQ, fluoroquinolone; EG, expert group; TB, tuberculosis; WHO World Health Organization; cfz, Clofazimine; RR-TB, rifampicin-resistant tuberculosis; lfx, levofloxacin; DR-TB, drug-resistant tuberculosis; Scr, short course regimen.
